# Robenacoxib shows efficacy for the treatment of chronic degenerative joint disease-associated pain in cats: a randomized and blinded pilot clinical trial

**DOI:** 10.1038/s41598-021-87023-2

**Published:** 2021-04-08

**Authors:** Derek Adrian, Jonathan N. King, Rudolph S. Parrish, Stephen B. King, Steven C. Budsberg, Margaret E. Gruen, B. Duncan X. Lascelles

**Affiliations:** 1 Translational Research in Pain (TRiP) Program, Department of Clinical Sciences, College of Veterinary Medicine, North Carolina State University, Raleigh, NC USA; 2 Elanco Animal Health, Companion Animal Development, Basel, Switzerland; 3 Elanco Animal Health, Companion Animal Development, Greenfield, IN USA; 4 Department of Small Animal Medicine and Surgery, College of Veterinary Medicine, University of Georgia, Athens, GA USA; 5 Behavioral Medicine, Department of Clinical Sciences, College of Veterinary Medicine, North Carolina State University, Raleigh, NC USA; 6 Comparative Pain Research and Education Centre, North Carolina State University, Raleigh, NC USA; 7 Department of Anesthesiology, Center for Translational Pain Research, Duke University, Durham, NC USA; 8 Thurston Arthritis Center, UNC School of Medicine, Chapel Hill, NC USA; 9 Present Address: Elanco Animal Health, Greenfield, USA; 10 Present Address: Vista Research LLC, Bath, ME USA

**Keywords:** Neuroscience, Somatosensory system, Pain, Physiology, Bone

## Abstract

The main objective of this pilot clinical trial was to evaluate outcome measures for the assessment of the nonsteroidal anti-inflammatory drug (NSAID) robenacoxib in cats with degenerative joint disease-associated pain (DJD-pain). Otherwise healthy cats (n = 109) with DJD-pain entered a parallel group, randomized, blinded clinical trial. Cats received placebo (P) or robenacoxib (R) for two consecutive 3-week periods. Treatment groups were PP, RR, and RP. Actimetry and owner-assessment data were collected. Data were analyzed using mixed-effects and generalized mixed-effects linear models. Activity data showed high within-cat and between-cat variability, and 82.4% of the values were zero. Compared to placebo, mean total activity was higher (5.7%) in robenacoxib-treated cats (p = 0.24); for the 80th percentile of activity, more robenacoxib-treated cats had a > 10% increase in activity after 3 (p = 0.046) and 6 weeks (p = 0.026). Robenacoxib treatment significantly decreased owner-assessed disability, (p = 0.01; 49% reduction in disability; effect size ~ 0.3), and improved temperament (p = 0.0039) and happiness (p = 0.021) after 6 weeks. More robenacoxib-treated cats were successes at 6 weeks (p = 0.018; NNT: 3.8). Adverse effect frequencies were similar across groups. Results identified suitable endpoints for confirmatory studies, while also indicating efficacy of robenacoxib in cats with DJD-pain.

## Introduction

Degenerative joint disease (DJD) and osteoarthritis (OA), types of chronic musculoskeletal disorder (CMSD)^1^, affect up to 90% of cats across all ages based on radiographic evidence^2^. OA is a type of DJD, and DJD refers to degeneration of both synovial and non-synovial joints. Most cats presenting for ‘OA-associated pain’ also have evidence of non-synovial joint deterioration and pain^2^, so the term DJD is probably better. Within-study data, based on examination of all cats by a single veterinarian unaware of the radiographic findings, indicates that 40% of cats with DJD had associated pain (DJD-pain) (unpublished data from^2^). No population-level estimates of the prevalence of DJD/OA or associated pain are available.

Establishing efficacy of analgesics in cats is challenging, notably in DJD-pain, since signs can be relatively subtle and caregiver assessments are prone to a very high placebo effect^3^. Current strategies to separate placebo effects from true therapeutic efficacy in DJD-pain include using validated owner questionnaires^4,5^, activity monitors (AMs) for objectively measuring movement or activity^6–8^, and novel study designs^6^.

Owner questionnaires for tracking pain, disability and response to treatment in cats, including the Client Specific Outcome Measures (CSOM) and Feline Musculoskeletal Pain Index (FMPI)^4,5,9^, have previously been developed and validated to varying degrees^10^. Whereas the CSOM allows owner selection and monitoring of three patient-specific activities over time, the FMPI requires owner assessment of patient impairment across 17 set activities, with two final questions addressing current and preceding pain levels. As has been recently summarized, none of the available questionnaires has been able to detect the presumed efficacy of nonsteroidal anti-inflammatory drugs (NSAIDs) in cats with DJD-pain in parallel design studies^10^.

Activity monitors have been fitted to collars or harnesses to record changes in acceleration associated with movement of the cat in its home environment, and have been used to discriminate between normal and affected animals in both research and client-owned populations and to monitor for treatment effects in analgesic studies^4,6–9,11–13^. However, previous work has shown that cats are inactive for the majority of the day (> 70% of the time), with peaks of activity occurring in the mornings and evenings, necessitating the use of complex models, time-restricted data sets, or other data partitions^14^ in order to assess differences between affected and non-affected animals, or to detect treatment effects.

Finally, novel study designs may reduce the high caregiver placebo effect^6^. Gruen and coworkers observed that detection of deterioration after masked discontinuation of an NSAID in cats might be a valuable proxy measurement of efficacy^6^, although positive results with this approach have not been replicated.

Currently, NSAIDs are the first line and mainstay of the treatment of the pain and inflammation that accompany musculoskeletal disease in humans and dogs^15^. However, there is rather limited information supporting the efficacy of NSAIDs in cats with DJD-pain, and there are presently no US FDA CVM-approved treatments for this indication in cats. It is possible that the limited information on efficacy of NSAIDs in cats reflects the difficulty of measuring chronic pain relief in cats.

The first NSAID approved for chronic use in cats (meloxicam in the EU^16^) has limited published evidence for efficacy in client-owned cats with naturally occurring DJD^4,5,9^. Additional data in a research colony of cats with naturally occurring OA indicated a positive effect of meloxicam in increasing night-time activity^11^. Robenacoxib is an NSAID with previously demonstrated efficacy in an induced (kaolin) model and clinical (acute musculoskeletal or surgical) pain^17–20^. Robenacoxib is presently registered in several countries (including Australia, Canada, Japan and the EU) for both acute and chronic use in cats with musculoskeletal disorders.

The main objective of this pilot study was to evaluate study designs and outcome measures to inform the design of a confirmatory field study. Additionally, we hypothesized that we would detect treatment-associated increases in activity (measured objectively by AMs) and improvement in subjective owner-perceived mobility impairment and pain, quality of life (QoL), temperament and happiness. We also hypothesized that we would observe no significant differences in adverse event (AE) rates between robenacoxib and placebo treatments.

## Results

**Figure 1:**
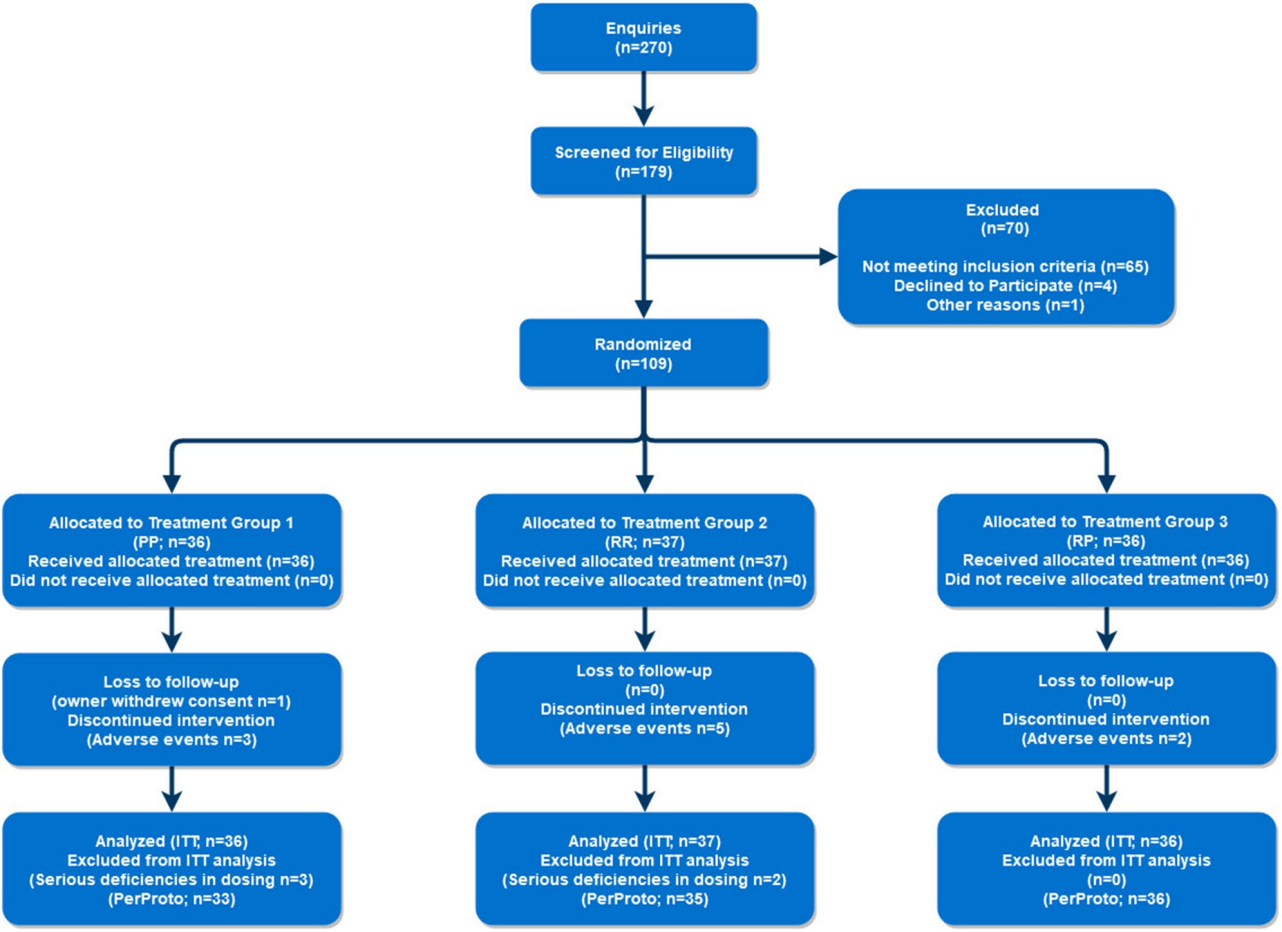
Patient recruitment and enrollment flowchart (CONSORT flowchart). *n*  number of patients; treatment groups: *P* placebo, *R* robenacoxib, *ITT* intent to treat analysis, *PerProto* per protocol analysis.

Recruitment and screening at the North Carolina State site occurred between 19 May 2014 and 31 July 2016, over approximately 115 weeks. Recruitment and prescreening at the University of Georgia site occurred between 31 March 2016 and 01 September 2016, over approximately 22 weeks, to supplement patient recruitment. Of approximately 270 enquiries, 179 cats were deemed eligible, scheduled for, and attended a screening appointment. Of the 179 cats screened, 70 were deemed ineligible for participation, and therefore 109 cats were enrolled (Fig. 1). Patient numbers were assigned according to study site and order of enrollment (e.g., LAS-01 for NC State, BUD-01 for University of Georgia).

Patient demographic data for all subjects (intent-to-treat (ITT) group, n = 109) are shown in Table 1. There were no significant differences between groups for body weight, age, or sex.

**Table 1:** Patient demographic data. Sex and breed are presented as count (percentage of treatment group total).

Characteristics	Group 1 (PP; n = 36)	Group 2 (RR; n = 37)	Group 3 (RP; n = 36)
**Sex**			
Male	17 (47.2%)	17 (45.9%)	12 (33.3%)
Female	19 (52.8%)	20 (54.1%)	24 (66.7%)
**Age (years)**			
Mean	11.6	11.1	11.8
SD	3.41	2.90	3.00
Range	3–17	3–15	2–17
**Body weight (kg)**			
Mean	5.70	5.34	5.76
SD	1.37	1.50	1.68
Range	3.89–9.19	3.16–10.7	3.25–9.75
**CKD status**			
IRIS Stages I or II	12 (33.3%)	5 (13.5%)	9 (25%)
No CKD	24 (66.7%)	32 (86.5%)	27 (75%)
**Breed category**			
American domestic medium hair	–	–	1 (2.78%)
Devon rex	–	–	1 (2.78%)
Domestic long hair	4 (11.1%)	5 (13.51%)	3 (8.33%)
Domestic medium hair	1 (2.8%)	1 (2.70%)	0 (0.00%)
Domestic short hair	21 (58.3%)	28 (75.7%)	29 (80.6%)
Maine coon	2 (5.56%)	1 (2.70%)	1 (2.78%)
Manx	2 (5.56%)	–	–
Oriental shorthair	–	1 (2.70%)	-
Persian	1 (2.78%)	–	–
Ragdoll	2 (5.56%)	1 (2.70%)	–
Rex mix	1 (2.78%)	–	–
Siamese	1 (2.78%)	–	1 (2.78%)
Siamese mix	1 (2.78%)	–	–

Treatment Groups 1–3—lettering designates the medications the patient received during the three treatment periods.

*P* placebo; *R* robenacoxib, *n* number of patients, *SD* standard deviation, *CKD* chronic kidney disease, *IRIS* International Renal Interest Society.

Efficacy data analyses are presented from the per-protocol analysis; the same conclusions were reached from the ITT analysis (data not shown). Five enrolled cats (LAS-11, 26, 37, 38, and 103) were excluded from the per-protocol analysis due to serious deficiencies in dosing (n = 5); in 4 cats these were due to adverse event (AE) associated early withdrawal (LAS-11, 26, 37, 38). Ten enrolled cats were removed from the study before Day 42. Reasons for early withdrawal included AEs (with gastrointestinal signs being most common), recurrence of historical disease processes, or withdrawal of owner consent (Table 2).

**Table 2:** Table detailing patients exiting the study early, with reason for withdrawal, study day of withdrawal, and treatment group.

Patient	Reason for withdrawal	Day (period) of withdrawal	Treatment group
LAS-11*	AE—neurological	Day 1 (T1)	2 (RR)
LAS-23	Withdrawal of owner consent	Day 19 (T1/T2)	2 (RR)
LAS-26*	AE—gastrointestinal—emesis	Day 4 (T1)	1 (PP)
LAS-28	AE—gastrointestinal—emesis	Day 23 (T2)	2 (RR)
LAS-37*	AE—gastrointestinal—emesis	Day 11 (T1)	2 (RR)
LAS-38*	AE—gastrointestinal—emesis	Day 15 (T1)	1 (PP)
LAS-40	AE—integumentary	Day 25 (T2)	3 (RP)
LAS-63	AE—gastrointestinal—emesis	Day 22 (T2)	1 (PP)
LAS-86	AE—gastrointestinal—emesis	Day 16 (T1)	3 (RP)
LAS-87	Recurrence of behavioral disorder—defecation	Day 22 (T2)	2 (RR)

*LAS* patient identification code corresponding to patients enrolled at North Carolina State University, *AE* adverse event, *T1* treatment period 1, *T2* treatment period 2, *P* placebo, *R* robenacoxib.

*Excluded from the PP analysis due to significant deficiencies in dosing.

### Primary outcome

The primary outcome measure was the change from baseline in mean hourly activity, assessed in all cats. There was very high variability between cats for measured activity, with individual mean hourly activity values ranging tenfold from 427 to 4793. Within-cat variability was also high; CVs within individual cats ranged from 253 to 598%. Analysis of total activity did not show a significant effect of treatment for either arithmetic or percent change from baseline (Table 3).

**Table 3:** Mean hourly activity data shown for the different analysis contrasts, presented as either arithmetic or percentage change from baseline.

Contrast	Arithmetic change			Relative change (%)		
	Estimate	SE	*P* value	Estimate	SE	*P* value
C1	116.4	80.4	0.15	5.65	4.78	0.24
C2	75.1	87.7	0.39	3.54	5.28	0.50
C3	− 23.2	60.3	0.70	1.25	3.38	0.71

Estimate = change from baseline in mean hourly activity counts with robenacoxib relative to placebo. Estimate = change from baseline in hourly activity counts with robenacoxib relative to placebo.

*C1* contrast 1 = treatment group PP compared against groups RR and RP following 3 weeks of treatment, *C2* contrast 2 = treatment group PP compared against group RR following 6 weeks of treatment, *C3* contrast 3 = treatment group RP compared against RR for change in activity between weeks 3 and 6 of treatment, *SE* standard error, *P* placebo, *R* robenacoxib.

*P values test whether the contrast is significantly different from zero.

### Secondary outcome measures

#### Activity—success/failure analysis

Using the 80th percentile activity data, over weeks 1–3, significantly more robenacoxib-treated cats versus placebo-treated cats increased their activity > 10% over baseline levels (42.3% versus 21.2% respectively; p = 0.046), and the same was true for weeks 1–6 (39.4% versus 32.8% respectively; p = 0.029).

#### Activity—partitioning for non-zero counts and dusk-to-dawn activity

For the cats in this study, 82.4% of AM one-minute epoch values were zero, supporting the analysis of only time periods when there was activity (non-zero counts). In the analysis of variance (ANOVA), the treatment × time-of-day interaction was significant (p < 0.05), justifying partitioning for day-time (08:00–20:00) and dusk-to-dawn-time (20:00–08:00) activity. Across all methods of data partitioning, cats receiving robenacoxib showed greater increases in activity compared to placebo (Table 4). Analysis of entire day non-zero activity and dusk-to-dawn activity showed greater, but non-significant, activity increases in robenacoxib-treated cats. Analysis of non-zero dusk-to-dawn values, revealed significant increases of approximately 11% after both 3 (C1) and 6 (C2) weeks of treatment with robenacoxib (p = 0.045 and p = 0.040, respectively). No significant deterioration (decrease in activity, C3) was detected across any partitioning of the data.

**Table 4:** Analysis of mean hourly activity with (‘all data’) and without (‘non-zero’) minutes when activity was zero.

Group/contrast	Arithmetic change			Relative change (%)		
	Estimate	SE	*P* value	Estimate	SE	*P* value
**Non-zero values, entire day**						
C1	141.2	82.3	0.089	6.61	4.46	0.14
C2	93.5	89.8	0.30	4.47	4.90	0.36
C3	− 40.5	61.7	0.51	0.33	3.15	0.92
**All data, dusk-to-dawn**						
C1	140.7	90.5	0.12	9.97	5.62	0.078
C2	165.7	98.8	0.097	11.6	6.10	0.060
C3	8.16	67.9	0.90	2.06	4.41	0.64
**Non-zero values, dusk-to-dawn**						
C1	158.9	94.8	0.097	10.70	5.29	0.045*
C2	172.9	103.9	0.099	11.9	5.74	0.040*
C3	− 2.41	68.7	0.97	1.74	4.18	0.68

Arithmetic change and relative change (percentage) for three contrasts that compare treatment groups. Results shown for non-zero activity across the whole day; all activity over the dusk-to-dawn time period; and non-zero activity over the dusk-to-dawn time period. Estimate = change from baseline in hourly activity counts with robenacoxib relative to placebo (positive values indicate greater activity when on robenacoxib).

*SE* standard error.

The contrasts were: C1 (contrast 1) = treatment group PP compared against groups RR and RP following 3 weeks of treatment; C2 (contrast 2) = treatment group PP compared against group RR following 6 weeks of treatment; C3 (contrast 3) = treatment group RP compared against RR for change in activity between weeks 3 and 6 of treatment. P = placebo; R = robenacoxib.

*Significance at 0.05 level.

#### Activity—within-cat analysis

**Figure 2:**
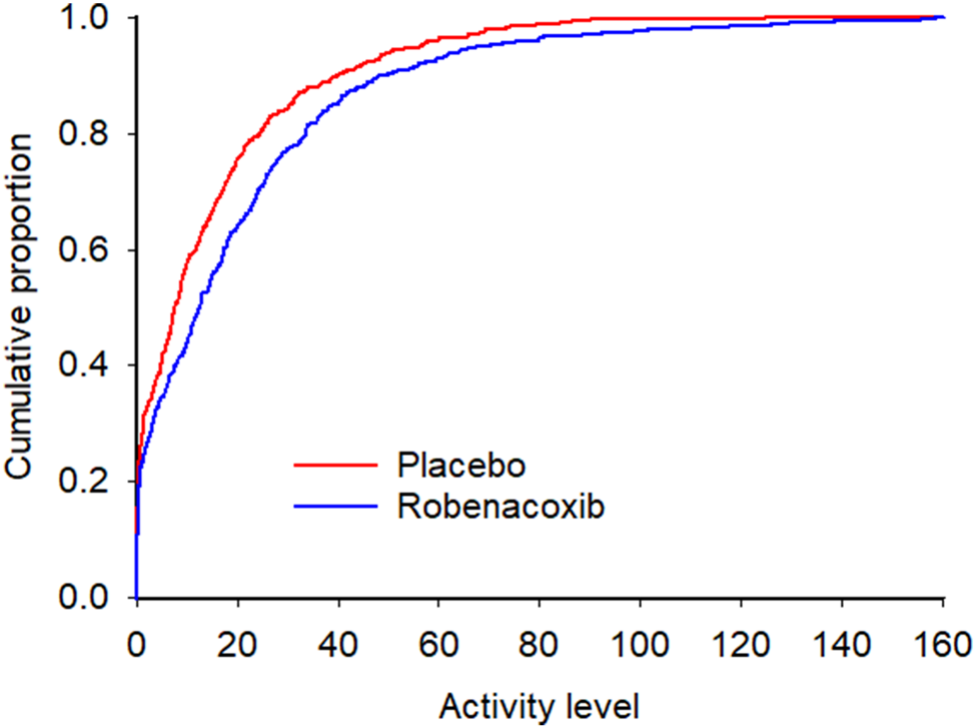
Example cumulative distribution function for a single cat (LAS-31), demonstrating a rightward shift of activity during treatment with robenacoxib, as compared against treatment with placebo. This indicates that the activity counts were higher while receiving robenacoxib. P values from the Kolmogorov–Smirnov test were < 0.0001 for the hypothesis that activity with robenacoxib > placebo, and 1.0 for placebo > robenacoxib. Analysis and graphical output performed using SAS software capabilities (Version 9, SAS Institute Inc., Cary, NC).

Group RP contained 35 evaluable cats for within-cat analysis using cumulative distribution function (CDF) analysis (example of CDF analysis in Fig. 2). More cats showed increases in activity with robenacoxib compared to placebo for all cats and the three subgroups (Table 5) with P values ranging from 0.021 to 0.059. Effects were statistically significant for entire-day non-zero activity (10:2 cats [28.6%:5.7%], p = 0.021) and dusk-to-dawn total activity (9:2 cats [25.7%:5.7%], p = 0.035) (Table 5). These ratios corresponded to number needed to treat (NNT) values of 4.4 and 5.0 cats, respectively.

**Table 5:** Analysis of within-cat activity data ( n = 35), showing the number and percentage of cats in which hourly activity counts were significantly higher during either placebo (P) or robenacoxib (R) treatment for each method of partitioning the data.

Group	n (% of total) of cats with activity R > P	n (% of total) of cats with activity P > R	P value	Difference in response rates (R – P)	NNT
Total mean hourly values, entire day	8 (22.9%)	2 (5.71%)	0.058	17.2%	5.83
Non-zero mean hourly values, entire day	10 (28.6%)	2 (5.71%)	0.021*	22.9%	4.37
Dusk-to-dawn mean hourly values	9 (25.7%)	2 (5.71%)	0.035*	20.0%	5.0
Non-zero mean hourly values, dusk-to-dawn	6 (17.1%)	1 (2.86%)	0.059	14.3%	7.0

*NNT* number needed to treat.

*Significance at 0.05 level, n = number

#### CSOM

CSOM total score demonstrated significantly greater improvement in robenacoxib-treated cats for both unadjusted (total scores) and baseline-adjusted (e.g., Day 42 score – Day 0 score) scores following six weeks of treatment [C2 = 1.40 (p = 0.010) and C2 = 0.94 (p = 0.044), respectively] (Table 6). This corresponds with effect sizes of 0.33 and 0.26, respectively. No other CSOM contrasts were significant.

**Table 6:** Analysis of owner based assessments of client specific outcome measures (CSOM) total score and Feline Musculoskeletal Pain Index (FMPI) score.

Owner assessment	Estimate	SE	t value**	Pr >|t|	Effect size
**CSOM total score**					
C1	0.49	0.52	0.95	0.34	0.09
C2	1.40	0.53	2.62	0.010*	0.33
C3	0.67	0.54	1.25	0.21	0.16
**CSOM total score adjusted for baseline**					
C1	0.23	0.46	0.50	0.62	0.05
C2	0.94	0.46	2.04	0.044*	0.26
C3	0.60	0.54	1.13	0.26	0.14
**FMPI score**					
C1	− 0.66	2.36	− 0.28	0.78	− 0.03
C2	4.65	2.58	1.80	0.075	0.18
C3	0.43	1.77	0.25	0.81	0.03
**FMPI score adjusted for baseline**					
C1	− 2.66	1.86	− 1.43	0.15	− 0.12
C2	0.75	1.96	0.38	0.70	0.04
C3	0.75	1.77	0.42	0.67	0.04

Estimates show the effect of robenacoxib relative to placebo.

*SE* standard error, *C1* contrast 1 = treatment group PP compared against groups RR and RP following 3 weeks of treatment, *C2* contrast 2 = treatment group PP compared against group RR following 6 weeks of treatment, *C3* contrast 3 = treatment group RP compared against RR for change in activity between weeks 3 and 6 of treatment. P = placebo; R = robenacoxib.

*Significance at 0.05 level.

**Student’s t statistic for testing whether the contrast equals zero; two-tailed test.

For success-failure rates based on CSOM, over weeks 1–3 there were no significant differences between the cats receiving robenacoxib versus placebo treated cats (57.6% versus 48.7% respectively; p = 0.35), but over weeks 1–6 significantly more cats dosed with robenacoxib versus placebo were deemed successes (81.2% versus 55.2%; p = 0.018). For the 6-week data, the NNT was 3.8.

CSOM baseline and post-treatment score analysis allowed for calculation of the reduction in pain and disability by treatment group (e.g., RR vs PP). The baseline score allowed for a calculation of how much improvement there could be, and the mean change allowed calculation of the actual improvement as a percentage as what was possible. After 6 weeks of treatment, in the PP group, the improvement of 2.28 points equated to a 30% reduction in disability, and in the RR group, the improvement of 3.53 points equated to a 49% reduction in disability, and an improvement with robenacoxib over placebo of 19%. The actual CSOM scores for each group at baseline, and at 3 and 6 weeks, and the change from baseline, are shown in Supplementary Table 1.

#### FMPI, quality of life (QoL), temperament and happiness assessments

No significant treatment effects on FMPI scores (nominal or adjusted) were detected (Table 6).

Analysis of temperament and happiness compared to before the most recent treatment showed significant improvement following 6 weeks of treatment with robenacoxib compared with placebo [odds ratio, OR, = 4.53 (p = 0.0039) and OR = 2.73 (p = 0.021), respectively], while all other comparisons (3 weeks for temperament and happiness; 3 and 6 weeks for QoL) failed to show significance (Table 7). The frequency distribution tables for temperament, happiness and QoL are shown in Supplementary Tables 2, 3 and 4 respectively.

**Table 7:** Results of owner-based assessments of quality of life (QoL), temperament, and happiness.

Owner assessment	Odds ratio	95% confidence interval		*P* value
**Quality of life compared to before most recent treatment**				
C1	1.17	0.47	2.94	0.73
C2	2.09	0.87	5.04	0.098
C3	2.33	0.59	9.19	0.22
**Temperament compared to before most recent treatment**				
C1	1.41	0.50	3.99	0.51
C2	4.53	1.65	12.5	0.0039*
C3	3.44	0.82	14.4	0.090
**Happiness compared to most recent visit**				
C1	1.61	0.64	4.06	0.31
C2	2.73	1.17	6.38	0.021*
C3	1.47	0.39	5.59	0.57

The odds ratios show the effect of robenacoxib relative to placebo.

*C1 *contrast 1 = treatment group PP compared against groups RR and RP following 3 weeks of treatment, *C2* contrast 2 = treatment group PP compared against group RR following 6 weeks of treatment, *C3* contrast 3 = treatment group RP compared against RR for change in activity between weeks 3 and 6 of treatment. The tests are effectively comparing the categorical response probability distributions. An odds ratio greater than 1 indicates a higher likelihood of better outcomes for the robenacoxib treatment. P = placebo; R = robenacoxib.

*Significance at 0.05 level.

### Safety measures

Adverse events were generally mild and self-limiting, typically involving the gastrointestinal tract (Table 8). None of the rates of occurrence of AEs were significantly different between cats receiving placebo or robenacoxib. The proportions of cats with pre-existing chronic kidney disease (CKD) experiencing at least one AE during the study were not significantly different between treatment groups (p = 0.88).

**Table 8:** Summary of the number of cats experiencing adverse events by clinical sign for both placebo and robenacoxib treatment.

Clinical sign	T1 placebo	T1 robenacoxib	*P* value	T2 placebo	T2 robenacoxib	*P* value
	n = 36	n = 73		n = 69	n = 34	
Emesis	7	10	0.57	2	4	0.091
Lethargy	5	3	0.11	2	1	> 0.99
Anorexia	4	3	0.22	0	1	0.33
Intestinal disorder NOS*	2	1	0.25	1	2	0.26
Pruritus	0	1	> 0.99	1	1	> 0.99
Inappropriate Urination	1	2	> 0.99	0	1	0.33
Diarrhea	0	1	> 0.99	3	0	0.55
Renal Insufficiency	0	0	N/A	2	2	0.60
Weight loss	1	0	0.33	1	2	0.25
Anemia NOS	0	1	> 0.99	2	0	> 0.99
Intestinal stasis	1	1	> 0.99	1	0	> 0.99

*T1* treatment period 1, *T2* treatment period 2, *NOS* not otherwise specified, *n* total number of cats receiving treatment in specified period. There were no significant differences in rates of adverse events occurrence between treatments.

No clinically relevant hematological, chemistry, or urinalysis differences between groups were observed. Several differences were observed between treatment groups at study exit. Select hematological, chemistry, and urinalysis data, including all statistically significant results, are available as Supplementary Tables 5, 6 and 7 respectively.

## Discussion

The study achieved its main objective of identifying suitable outcome measures for testing the efficacy of the NSAID robenacoxib in cats with DJD-pain. Additionally, collectively, the data reported in this study support the hypothesis that cats receiving robenacoxib would show increases in AM-measured physical activity when compared to placebo, and the hypothesis that robenacoxib treatment would be associated with owner-assessed decreases in pain and disability, and improvements in temperament and happiness. However, these improvements were only seen after 6 weeks of treatment, and not after 3 weeks of treatment.

As has been reported previously for other NSAIDs^4,5^, it was confirmed that between-cat comparison of total activity was not sufficiently discriminating between robenacoxib and placebo, but that analysis of higher levels of activity (either non-zero counts or a pre-specified high percentile of activity), dusk-to-dawn activity and/or within-cat analyses were more sensitive. In addition, owner subjective assessments of disability, temperament and happiness, but not the version of the FMPI employed in this study, appeared to detect treatment benefits. In our study, 82.4% of activity counts were zero, indicating no measurable activity. This information justified partitioning of the data to analyze the more active times. Analysis of partitioned data showed greater activity with robenacoxib versus placebo for dusk-to-dawn activity and non-zero activity. Recent data demonstrate that higher levels of activity are more impacted in cats with DJD-pain and NSAIDs appear to preferentially positively affect these higher levels, or latent states (manuscript in review). Consistent with these observations, in our study robenacoxib produced a significant increase in activity by ≥ 10% in the 80th percentile of activity values.

Between-cat variability is very high for activity^4,14^, supporting the suggestion that within-cat analysis of data may be superior to between-cat analysis (as used in our primary analysis). In our study, between-cat variability for activity was very high and within-cat variability was lower but still high. Due to the study design, within-cat analysis of our data was restricted to the analysis of the RP group of 35 cats, which sequentially received placebo (baseline), robenacoxib and then placebo. Use of a full cross-over design would allow investigators to minimize variability and take advantage of the increased power associated with each cat being compared to itself.

Significantly better outcomes with robenacoxib compared to placebo were obtained with three of the owner-based outcome measures—CSOM, temperament and happiness assessments. We failed to detect any significant differences between groups using the FMPI questionnaire. The FMPI consists of set questions, and overall, in its current form, it does not appear to be as sensitive to treatment effects as the individually tailored CSOM^10^. The authors (BDXL, MEG) have recently been revising the FMPI. Treatment effects on CSOM (actual scores and success-failure), temperament and happiness were significantly improved following 6 weeks of treatment with robenacoxib, but not after three. This lag may be due to delays in owners noticing or learning to detect behavioral changes, time required for cats to un-learn their learned avoidance or fear of activities, or other unknown factors. The lag time may also be due to pharmacokinetic factors. However, this unlikely because robenacoxib should achieve steady concentrations at sites of inflammation within a few days and exhibits no changes in pharmacodynamic action with time^19^.

For activity, we observed increases of approximately 5% and 10% with robenacoxib over placebo for total and non-zero or dusk-to-dawn activity, respectively. These values compare favorably with the previously reported increase of 3.32% (non-significant) over placebo in total activity in cats with DJD receiving the NSAID meloxicam for three weeks^4^. However, Gruen et al.^4^ evaluated meloxicam administered at 0.035 mg/kg daily—less than the maintenance dose of 0.05 mg/kg daily approved in the EU. No studies examining activity changes in humans with OA who are administered analgesics have been performed to help put these changes into context. Overall, little is known about what constitutes a clinically relevant change in activity. Additionally, it is clear from these data that a relatively small proportion of cats significantly increased overall activity. Even if one accepts that ‘activity’ or ‘movement’ is improved in cats suffering chronic DJD-pain which are treated with an effective analgesic, much remains to be learned about how to analyze and interpret such data.

In humans, reductions of pain of 15%, 33%, and 50% were reported to correlate with the minimal clinically important difference (MCID), ‘much better’ improvement^21^, and ‘very much improved’^22^, respectively. The MCID of veterinary species is unknown, but our data show a 49% reduction in disability (CSOM) after 6 weeks of robenacoxib treatment which would equate to ‘very much improved’ in human medicine. Standardized effect sizes are another accepted way of comparing efficacy of treatments across studies. Baseline-adjusted CSOM scores indicated an effect size (ES) (for treatment over placebo) of 0.26 when comparing groups PP and RR following 6 weeks of treatment. While no ES data for any outcome measures are available for dogs or cats with DJD treated with NSAIDs, the ES for efficacy of NSAIDs in hip and knee OA in humans is approximately 0.3 when based on high-quality trials^23, 24^. Another method used to compare the efficacy of treatments across studies is number needed to treat (NNT). Based on the within-cat analysis of change in activity while on placebo and robenacoxib, the NNT values compare favorably with those for NSAIDs used in humans to treat chronic pain (between 3 and 13 depending on the criteria for success)^25^. In contrast to NNT for chronic pain, the NNTs for NSAID alleviation of acute pain tend to be lower^26^.

The current data did not support the hypothesis that the study would detect a deterioration in outcome measures following discontinuation of robenacoxib, as compared against cats which continued to receive the medication. While a single previous study has reported detection of deterioration after withdrawal of the NSAID meloxicam^6^, there are no published reports of this approach having been replicated. Our study may have been insufficiently powered to detect deterioration and deterioration was only assessed after 3 weeks treatment; a greater effect might be detected after longer treatment.

Our CSOM data confirm previous reports of a significant caregiver placebo effect in feline chronic pain studies. Placebo effect sizes calculated using the following equation (Cohen’s d for the placebo group alone):

$$(Mean{ }Score_{Placebo} { } - { }Mean{ }Score_{baseline} )/\left( {Pooled{ }standard{ }deviation} \right)$$

were 1.40 and 1.68 following 3 and 6 weeks of administration of placebo, respectively. These values are at the top end of the placebo effect sizes reported by Gruen et al.^3^, and research is needed to understand what drives this placebo effect, and how to mitigate or control it in clinical studies. High placebo effects make it difficult to detect positive treatment effects.

The safety data we report corroborate the previously published clinical safety of robenacoxib in OA-affected cats (with and without CKD)^27^. Most AEs were self-limiting and did not require medical intervention. Furthermore, cats with International Renal Interest Society (IRIS) stage 1 or 2 CKD were no more likely to experience an AE, which is important given the high prevalence of both CKD and DJD in cats^28^. However, the study design was optimized for efficacy rather than safety assessment.

This study has several limitations. There is no easy definitive way to diagnose DJD-pain in cats, so the approach to diagnosis was based on what has been established through clinical research and published in the literature, and used a combination of owner assessment and veterinarian assessment, as well as radiography. The current study did not attempt to grade DJD, or grade the impact of DJD-pain. The disease of DJD could be graded by assigning severity scores to radiographs: grading the impact of DJD-pain on the whole individual has not been described in veterinary medicine other than the use of owner assessments.

The observed high between-cat variability relative to sample size is likely responsible for the lack of statistical significance in several comparisons. The within-cat analysis of activity data was based on CDFs of a single treatment arm (RP) rather than a full crossover design, and the data partitions used may not be equally applicable to all cats. For example, dusk-to-dawn was predefined as 20:00 to 08:00 and individual cats differ from each other in their bimodal activity distribution over the 24-h period of a day. Nonetheless, the current approach enabled us to evaluate each cat in a binary sense as having or not having a significant improvement under robenacoxib compared to placebo. However, all of these cats received robenacoxib and then placebo, and the carry-over effect of robenacoxib on the placebo phase is unknown.

The outcomes of QoL, temperament and happiness have not been previously reported, and the questionnaires we used have not been validated either as measures of these factors, or for responsiveness validity. No attempt was made to explain the terms "QoL", "happiness" or "temperament" to owners, but rather allow each owner to interpret the terms themselves. These assessments were included because of our (BDXL, MEG) increasing experience and belief that many dimensions are impacted by pain^10^, and objective accelerometry and clinical metrology instruments based on activity and mobility (e.g. CSOM, FMPI) do not capture all these dimensions, especially the affective dimensions. Previous research has identified some of the aspects that owners consider are important to QoL^29^, confirming that non-active aspects are important to owners. Further research should investigate what the terms "happiness" and "temperament" actually mean to owners. Regardless, the use of these novel assessments revealed positive treatment effects in the current blinded, placebo-controlled study.

Our findings may not be generalizable to the entire population of cats with OA/DJD-pain. The majority of enrolled cats were from a single study site, presenting a geographical bias. Furthermore, while some comorbidities were allowed by the inclusion/exclusion criteria, cats were required to be overall healthy, meaning findings may be different in the general population of cats with mobility impairment. Lastly, inclusion criteria required that cats be moderately to severely impaired, meaning that results may be different in cats with milder impairment.

In conclusion, results of this pilot study identified suitable outcome measures and approaches to data partition for testing the efficacy of the NSAID robenacoxib in cats with DJD-associated pain in a subsequent confirmatory study. Additionally, overall, we detected significant robenacoxib-associated improvements in both objective and subjective outcome measures following 6 weeks of treatment. However, many data were not significant, and activity data were only significant for particular time periods of the day. No deterioration following masked discontinuation of robenacoxib was detected, possibly reflecting the lack of a significant effect at 3 weeks. While significant effects on patient temperament and happiness were detected, these measures have not been previously validated, and no explanations were given to owners as to what these terms meant. Notwithstanding these comments, the level of evidence for the efficacy of robenacoxib derived from this study is, in our view, at least as good as that published for other NSAIDs in cats with DJD-associated pain.

## Materials and methods

All procedures performed in this study were approved by the relevant North Carolina State University Institutional Committees. All methods were carried out in accordance with relevant guidelines and regulations. This study was approved by the Animal Care and Use Committees at North Carolina State University College of Veterinary Medicine (IACUC protocol 14-009-O), University of Georgia College of Veterinary Medicine (IACUC protocol CR-447) and Novartis Animal Health. Written owner consent was provided for each case before pre-enrollment after verbal discussion of the study. This manuscript was prepared after consultation of the CONSORT checklist for reporting of parallel-group randomized trials^30^.

### Study design

**Figure 3:**
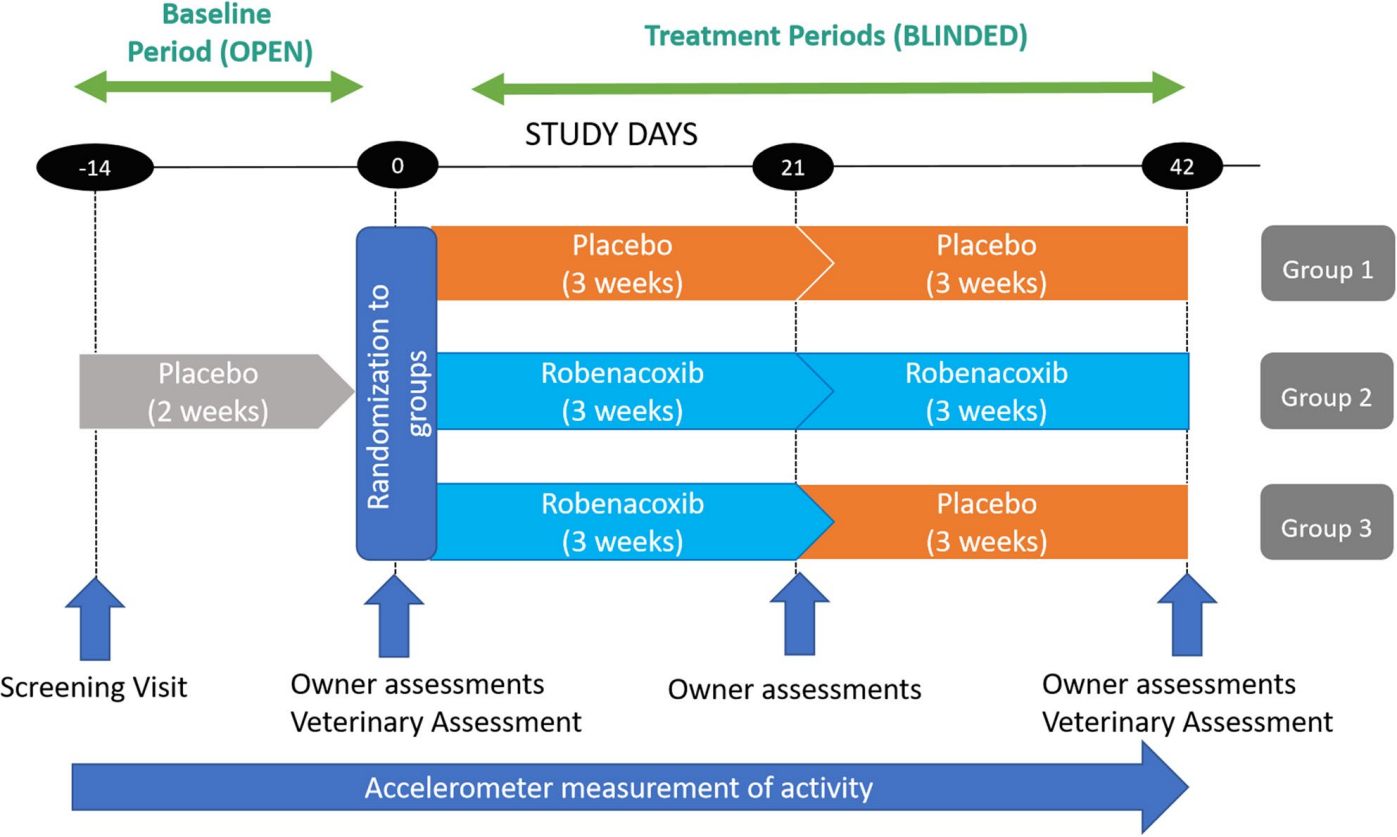
Study design and timeline. The blinded study started at Day 0. Screening (examinations, owner questionnaires, bloodwork, fitting the cat with an activity monitor) occurred 2 weeks (14 days) prior to Day 0. Cats were examined in the clinic, and owners completed assessments at Day 0 and Day 42. Owners visited the clinic alone to complete assessments on Day 21. Treatment period 1 (T1) was over days 0–21 (3 weeks); treatment period 2 (T2) was over days 22–42 (3 weeks).

This study was conducted in compliance with Good Clinical Practices and was a double-blind, placebo-controlled, randomized study with 3 parallel arms (groups) (Table 9). All three groups had an open (unblinded) baseline (BL) period, and then two (blinded) treatment periods of three weeks each (Placebo-Placebo (PP); Robenacoxib-Robenacoxib (RR); Robenacoxib-Placebo (RP)) (Fig. 3). Study days were defined in relation to the first day of blinded treatment (designated Day 0), with Day − 14, Day 0, Day 21 (3 weeks), and Day 42 (6 weeks) involving site visits by the owner and cat, except for Day 21 when just the owner visited (Fig. 3). A minimum sample size of 20 cats per group was estimated based on previous work with accelerometers^5^. Using previous data from an NSAID study, a treatment-placebo group difference in hourly activity of 75.2 and a SD of 92.6, the power was calculated to be 80% with group sizes of 25 cats per group and 90% with 33. Cats were randomized in a 1:1:1 ratio, via permuted block randomization with block size of 3, according to pre-determined randomization tables (generated by the original study statistician using SAS (Version 9, SAS Institute Inc., Cary, NC)) for each site. Medication dispensing was performed by pharmacy personnel not involved in patient assessment or data collection. All people involved in the study were blinded to the treatments until after the database was locked, with the exception of one sponsor representative (SBK).

**Table 9:** Treatment group designations and treatments by period.

Group and treatment sequence	2-week baseline period (non-blinded)	3-week treatment period 1 (T1^a^)	3-week treatment period 2 (T2^a^)
1 (PP)	Placebo	Placebo	Placebo
2 (RR)	Placebo	Robenacoxib	Robenacoxib
3 (RP)	Placebo	Robenacoxib	Placebo

*P* placebo, *R* robenacoxib.

^a^Treatment periods T1 and T2 were blinded.

Cats were client-owned with naturally occurring DJD-associated pain and owner-assessed mobility impairment. Cats remained in the care of their owners at home throughout the study except for visits to the clinic. Subjects were recruited using advertising to owners and veterinarians. All study-related costs, including to the owners, were covered by the sponsor, as were recruitment incentives.

Patient screening and data collection were performed at both the North Carolina State and University of Georgia study sites. Cats were screened for eligibility and enrolled in a similar manner to previous DJD-associated pain studies in cats performed by the authors^4^. On the day of screening (Day − 14), cats underwent physical, orthopedic, and neurological exams. Blood and urine samples were obtained for hematology, serum biochemistry, urinalysis with sedimentation, and serum T4 analysis at an external laboratory (Antech Diagnostics, Southaven, MS). Complete axial and appendicular orthogonal radiographs were obtained under sedation and were reviewed by a board-certified veterinary radiologist and the lead investigator (BDXL).

#### Inclusion/exclusion criteria

Cats were required to have at least moderate owner-assessed mobility or activity impairment (CSOM < 6, see below), evidence of pain during orthopedic evaluation of at least two joints or spinal segments, with radiographic changes associated with DJD in at least two joints or spinal segments identified to have pain. The same investigator at each site performed orthopedic pain assessments. Cats were also required to be at least 1 year of age, between 2.5 and 12.0 kg in weight (to allow dosing with available tablets), and to be generally healthy and not currently receiving analgesic or anti-inflammatory medications (including potential analgesics). Cats with controlled diabetes, hyperthyroidism, or stable CKD (international renal interest society (IRIS) stages 1 and 2) were allowed to participate. Cats were required to be primarily indoor only to avoid potential AM loss.

Cats meeting these criteria were pre-enrolled and entered an approximately 2-week acclimation/baseline period. Activity measurements over BL provided the baseline AM data, although only the 7 days prior to Day 0 were used in efficacy analyses in order to try to avoid the confounding effects of the screening visit, sedation medications, and acclimation to the collar or harness. This BL period, during which known placebo was administered, also served as an additional screening step to determine an owner’s ability to administer medication (unmasked placebo, see below) and keep daily records of dosing and patient behaviors.

Following BL, cats were randomized and entered into the blinded portion of the study (by DA or SB), and allocated to one of three treatment groups (Table 9) to receive a daily minimum oral dosage of 1 mg/kg (range 1–2.4 mg/kg) of robenacoxib (the commercially available formulation (Onsior) supplied as 6 mg tablets) or an equivalent number of placebo tablets. Both the robenacoxib and placebo tablets and their packaging appeared identical and were supplied in packaging identical to the commercially available formulation (Onsior) in aluminum blister pack cards of 6 tablets each.

Owners were instructed to administer the tablet(s) directly into the cat’s mouth or mixed with a small amount of food (one third or less of the daily food ration).

### Outcome measures

Although this was a pilot study, primary and secondary outcome measures were pre-defined in the protocol.

#### Primary outcome measure

The primary outcome measure was the change from baseline in mean hourly activity as measured by the AMs. Physical activity was measured on a per-minute basis using the Actical AM device. Devices were configured as previously described^4^, with the epoch set at 1 min. The AM device was placed on a non-breakaway collar and placed upright on the patient’s ventral neck.

Activity values within each hour were summed for use in statistical analysis, and mean hourly activity levels were computed for each cat over the last seven days of BL (“Day 0 mean”) and over the first 20 days of the T1 and T2 periods separately (“Day 21 mean” and “Day 42 mean”, respectively). Days 0, 21 and 42 were ‘travel’ days for the cat and were not included in the analyses.

#### Secondary outcome measures

Secondary outcome measures were additional analyses of the activity data plus owner-based subjective assessments of mobility impairment and pain, QoL, temperament and happiness.

#### Secondary outcome measures—activity

(a) Activity—success/failure analysis:

Using the 80th percentile of activity data (approximating the time that most cats are active), the number of cats in each group that increased their activity by 10% or greater over baseline levels was calculated, and groups compared for weeks 1 to 3, and weeks 1 to 6.

(b) Activity—partitioning for non-zero counts and dusk-to-dawn activity:

Previous work had shown minimal changes in mean daytime activity in cats given meloxicam compared to placebo over 3 weeks^4^. In addition, there are data indicating increases in night-time activity but not mean daytime activity in research cats with OA which were administered meloxicam^11^, and also that most cats are inactive for the majority of the day (> 70% of the time)^14^. Based on a review of previous data^4^, the data set was also partitioned into "daytime" (defined as 08:00–20:00) and "dusk-to-dawn" (defined as 20:00–08:00) observations. These data subsets allowed for combinations of mean hourly total values and mean hourly non-zero values, for the entire day, daytime, or dusk-to-dawn comparisons.

(c) Activity—within-cat analysis:

Previous studies have shown the value of a cross-over design, since between-cat variability is high in cats with DJD-pain^4,5,9^. In our study, group 2 (RP) cats received robenacoxib then placebo in T1 and T2, respectively, allowing for within-cat analysis of activity. Within-cat analysis of activity used the average per-minute values on an hourly basis.

#### Secondary outcome measures—owner subjective assessments

Owner assessments included the CSOM and FMPI questionnaires (available at: https://cvm.ncsu.edu/research/labs/clinical-sciences/comparative-pain-research/clinical-metrology-instruments/).

The CSOM required owners to select three activities (either from the FMPI or self-generated) that their cat had difficulty performing, instructing the owner to rate the cat’s ability to perform the task over the past week. Owners assigned an integer score from 0 to 4 (0 = impossible, 4 = no problem) for each activity, with a CSOM total score ranging from 0 to 12. Data were evaluated based on actual scores, change from baseline, and also on a success-failure basis. Success was defined for each cat as an increase in total CSOM score of 2 or greater (decrease in disability), with no individual question score getting worse. A change in CSOM score greater than or equal to 2 has been used in dogs^31^ and was considered clinically relevant.

For the FMPI questionnaire, owners rated their cat’s ability to perform 17 set activities on an integer scale of 0 to 4 (0 = not at all, 4 = normal); with a total FMPI score ranging from 0 to 68. Scores were adjusted if questions were unanswered or not-applicable by taking the sum of scores for answered questions and multiplying by 68/(4 times the number of questions answered). Two final questions assessed cat pain levels on a visual analogue scale from 0 to 100 mm, with 0 representing “severe pain” and 100 representing “no pain”. The owner’s mark on the line was measured and converted to a number and analyzed separately.

At each study visit, QoL, temperament, and happiness in comparison to before the initiation of treatment (the study) were rated by the owner. Each outcome was rated on a 5-point Likert-type scale. Both QoL and temperament compared to before were rated from “much worse” to “greatly improved”. Finally, happiness was rated from “much more unhappy” to “much more happy” compared to before. Owners were not provided any descriptors of QoL, temperament or happiness; they were instructed to complete the form based on how they interpreted the terms. This form is available in Supplementary Figure 1. The responses were summarized into frequency distributions for analysis.

#### Safety assessments

Owners were instructed to immediately report any AEs to the investigators, and owners were proactively questioned about AEs (including anorexia, diarrhea, lethargy, vomiting) at each visit. AEs were defined as any observations in the cat that were deemed unfavorable and unintended that occurred during the study period, whether they were considered treatment-related or not. Serious AEs (SAE) were defined as AEs that were fatal or life-threatening, required veterinary intervention, or were considered clinically serious by the investigators. Owners were required to bring their cat to the study site or local veterinarian if an SAE was suspected.

### Statistical methods

Data were analyzed on both ITT and per-protocol data sets. The ITT data set was used for safety analysis and consisted of data from all randomized animals that received at least one dose of study medication after Day 0. The per-protocol data set was used for efficacy analysis. The statistical model used was repeated measures ANOVA. A mixed-effects linear model was used for continuous response variables and a generalized mixed-effects linear model was used for categorical response variables, both of which involved, as fixed effects, sequence group, treatment period, treatment, and treatment × period, as well as site and site × treatment as random effects. These were implemented using the MIXED and GLIMMIX procedures of SAS software, respectively. Most analyses used baseline-adjusted responses (arithmetic or relative change from baseline). Three linear contrasts (termed C1, C2, and C3) were evaluated using the group- and period-specific least-squares means. C1 compared the placebo (P) to robenacoxib (R) during T1 (i.e., over the first 3 weeks). C2 compared the placebo to robenacoxib responses during T1 and T2 combined (i.e. over all 6 weeks). Finally, C3 compared changes in mean responses after discontinuation of robenacoxib in T2 (i.e., deterioration), using groups 2 and 3. These are summarized as:

$$\begin{gathered} {\text{C1 }} = \, 0,{\text{ where C1 }} = \, \left( {{\text{Rp }} + {\text{ Rr}}} \right)/{2 }{-}{\text{ Pp}};{\text{ at 3 weeks}} \hfill \\ {\text{C2 }} = \, 0,{\text{ where C2 }} = \, \left( {{\text{Rr }} + {\text{ rR}}} \right)/{2 } - \, \left( {{\text{Pp }} + {\text{ pP}}} \right)/{2};{\text{ at 6 weeks}} \hfill \\ {\text{C3 }} = \, 0,{\text{ where C3 }} = \, \left( {{\text{Rp }}{-}{\text{ rP}}} \right) \, - \, \left( {{\text{Rr }}{-}{\text{ rR}}} \right);{\text{ at 6 weeks following discontinuation of R at 3 weeks}} \hfill \\ \end{gathered}$$

where upper case letters denote the sequence-treatment period means being used. The estimated values of the contrasts indicate the magnitude and direction (robenacoxib versus placebo) of the associated effects. Rp, Rr, and Pp represent means across the three arms for the T1 period. rP, rR, and pP correspond to means for the T2 period. The contrasts are linear functions of these means reflecting T1-level effects and T1 + T2 level effects.

All analyses were conducted at a two-sided 0.05 level of significance. No adjustments for multiple comparisons were made in order not to inflate the type II error rate; the study was a pilot and many of the efficacy endpoints may be correlated (i.e., not independent) and therefore adjustment of the P value using techniques such as the Bonferroni method might be overly conservative.

For the activity endpoint (i.e., mean hourly activity level within treatment periods), the response variables were the arithmetic and the relative (percent) change from baseline. For baseline values, the last 7 days of the BL period were used, thus allowing for acclimation during the initial portion of the BL period. The model described above was applied for full-day data and for data partitioned by time-of-day periods.

A generalized linear model with logit link was used to test the success-failure rates for the increase in activity within the 80th percentile of hourly activity, setting the threshold for success as an increase of at least 10% over the baseline percentile.

For within-cat analysis of activity, the cumulative distribution functions (CDFs) of hourly activity data for both treatment periods were examined and tested for significant differences. The nonparametric two-sample Kolmogorov–Smirnov test statistics (D_−_ and D_+_) of the maximum observed differences between the robenacoxib and placebo empirical distribution functions were computed and the associated *P* values were calculated for each cat individually. In this study, D_-_ corresponds to stochastically higher activity levels for robenacoxib compared to placebo, while D_+_ corresponds to the opposite case. The proportion of cats demonstrating significantly higher levels of activity under robenacoxib (*P* < 0.05) was compared with the proportion that had significantly higher activity under the placebo using McNemar’s test. The NNT i.e. the estimated number of cats that need to receive robenacoxib in order for one cat to benefit, on average, was calculated as the inverse of the difference between the proportions of cats improved with robenacoxib compared with placebo.

CSOM data were analyzed using a mixed linear model for total score (unadjusted) and the change in total score relative to baseline (adjusted). Additionally, success-failure analysis was based on a decrease in CSOM total scores of 2 units or more defining ‘deterioration’ (as previously reported^6^), and the proportions were compared between the RR and RP groups using a generalized linear model (logit link).

A mixed effects linear model was used to analyze FMPI score^4^. Standardized effect sizes (ES) were calculated for CSOM and FMPI endpoints using the equation: [mean of treatment group minus the mean of the control group]/[pooled standard deviation].

Happiness, temperament and QoL responses were analyzed using a generalized mixed linear model for ordered categories, which modeled the probabilities of levels of the response variable having lower ordered values (cumulative logit link). For these analyses, ORs were calculated and expressed so that values greater than 1 indicated the occurrence of more positive outcomes associated with the robenacoxib treatments.

Frequency data (e.g., for AEs and changes in clinical pathology variables) were analyzed using Fisher’s exact test. Body weight, complete blood count (CBC), clinical chemistry and urinalysis variables were analyzed using ANOVA.

### Ethics declarations and approvals for animal experiments

This study was approved by the Animal Care and Use Committees at North Carolina State University College of Veterinary Medicine (IACUC protocol 14-009-O), University of Georgia College of Veterinary Medicine (IACUC protocol CR-447) and Novartis Animal Health. Approval by University IACUC committees is only granted if studies follow Animal Welfare Act guidelines. Written owner consent was provided for each case before pre-enrollment after verbal discussion of the study. This manuscript was prepared after consultation of the CONSORT checklist for reporting of parallel-group randomized trials.

## Supplementary Information

Supplementary Information.

## Data Availability

The datasets generated during and/or analyzed during the current study are available from the corresponding author on reasonable request.
