# Supplementary material for: Robenacoxib shows efficacy for the treatment of chronic degenerative joint disease-associated pain in cats: a randomized and blinded pilot clinical trial
Source: Sci Rep. 2021 Apr 8;11:7721. doi: 10.1038/s41598-021-87023-2 (PMC8032665; doi:10.1038/s41598-021-87023-2)
Supplement: Supplementary file 1 — Supplementary Information. [file 41598_2021_87023_MOESM1_ESM.docx]

SUPPLEMENTARY MATERIALS

**Title:** Robenacoxib shows efficacy for the treatment of chronic musculoskeletal disorder-associated pain in cats: A randomized and blinded pilot clinical trial

**Authors:** Derek Adrian^a,#^, Jonathan N. King^b^, Rudolph S. Parrish^c,^^, Stephen B. King^c^, Steven C. Budsberg^d^, Margaret E. Gruen^a,e^, B. Duncan X. Lascelles^a, f, g, h *^

**Affiliations:**

^a^ Translational Research in Pain (TRiP) Program, Department of Clinical Sciences, College of Veterinary Medicine, North Carolina State University, Raleigh, NC

^b^ Elanco Animal Health, Companion Animal Development, Basel, Switzerland

^c^ Elanco Animal Health, Companion Animal Development, Greenfield, IN

^d^ Department of Small Animal Medicine and Surgery, College of Veterinary Medicine, University of Georgia, Athens, GA

^e^ Department of Clinical Sciences, College of Veterinary Medicine, North Carolina State University, Raleigh, NC

^f^ Comparative Pain Research and Education Centre, Department of Clinical Sciences, College of Veterinary Medicine, North Carolina State University, Raleigh, NC

^g^ Center for Translational Pain Research, Department of Anesthesiology, Duke University, Durham, NC

^h^ Thurston Arthritis Center, UNC School of Medicine, Chapel Hill, NC

#DA is currently employed by Elanco Animal Health

^RSP is currently associated with Vista Research LLC

***** Corresponding author

[dxlascel@ncsu.edu](mailto:dxlascel@ncsu.edu)

| **CSOM Total Score by Sequence and Period** | | | | | | | | |
| --- | --- | --- | --- | --- | --- | --- | --- | --- |
|  | | | **Baseline** | | **Treatment** | | **Change from Baseline** | |
| **Group** | **Treatment Period** | **N** | **Mean** | **Std Error** | **Mean** | **Std Error** | **Mean** | **Std Error** |
| PP | T1 | 33 | 4.45 | 0.23 | 6.30 | 0.43 | 1.85 | 0.36 |
|  | T2 | 32 | 4.44 | 0.24 | 6.72 | 0.41 | 2.28 | 0.36 |
| RR | T1 | 33 | 4.85 | 0.28 | 7.36 | 0.40 | 2.52 | 0.34 |
|  | T2 | 32 | 5.00 | 0.28 | 8.53 | 0.40 | 3.53 | 0.42 |
| RP | T1 | 36 | 4.56 | 0.23 | 6.22 | 0.42 | 1.67 | 0.37 |
|  | T2 | 34 | 4.53 | 0.24 | 6.59 | 0.47 | 2.06 | 0.40 |

Supplementary Table 1: The actual CSOM scores for each group at baseline, and at 3 (T1) and 6 (T2) weeks, and the change from baseline. P = placebo; R = robenacoxib; N = number of animals; Std Error = standard error; T1 = end of 3 week treatment period; T2 = end of 6 week treatment period.

| **Temperament Compared to Before Recent Treatment** | | | | | | | | | |
| --- | --- | --- | --- | --- | --- | --- | --- | --- | --- |
|  | **PP** | | | **RR** | | | **RP** | | |
| **Response** | **BL** | **T1** | **T2** | **BL** | **T1** | **T2** | **BL** | **T1** | **T2** |
| SLIGHTLY WORSE | 0 | 1 | 1 | 1 | 1 | 2 | 1 | 0 | 3 |
| NO CHANGE | 32 | 17 | 20 | 25 | 8 | 5 | 32 | 23 | 22 |
| SLIGHTLY IMPROVED | 1 | 11 | 8 | 8 | 19 | 15 | 3 | 9 | 6 |
| GREATLY IMPROVED | 0 | 4 | 3 | 1 | 5 | 10 | 0 | 4 | 3 |

Supplementary Table 2: Frequency distributions of responses for ‘Temperament’. Frequencies provided for each group at each time point. P = placebo; R = robenacoxib; N = number of animals; Std Error = standard error; T1 = end of 3 week treatment period; T2 = end of 6 week treatment period.

| **Happiness Compared to Before Recent Treatment** | | | | | | | | | |
| --- | --- | --- | --- | --- | --- | --- | --- | --- | --- |
|  | **PP** | | | **RR** | | | **RP** | | |
| **Response** | **BL** | **T1** | **T2** | **BL** | **T1** | **T2** | **BL** | **T1** | **T2** |
| MUCH MORE UNHAPPY | 0 | 1 | 0 | 0 | 1 | 0 | 0 | 0 | 1 |
| SLIGHTLY MORE UNHAPPY | 1 | 2 | 2 | 1 | 1 | 2 | 2 | 2 | 2 |
| NO CHANGE IN HAPPINESS | 31 | 16 | 18 | 25 | 7 | 9 | 30 | 20 | 21 |
| SLIGHTLY MORE HAPPY | 1 | 10 | 7 | 7 | 19 | 12 | 4 | 9 | 4 |
| MUCH MORE HAPPY | 0 | 4 | 5 | 2 | 5 | 9 | 0 | 5 | 6 |

Supplementary Table 3: Frequency distributions of responses for ‘Happiness’. Frequencies provided for each group at each time point. P = placebo; R = robenacoxib; N = number of animals; Std Error = standard error; T1 = end of 3 week treatment period; T2 = end of 6 week treatment period.

| **QoL Compared to Before Recent Treatment** | | | | | | | | | |
| --- | --- | --- | --- | --- | --- | --- | --- | --- | --- |
|  | **PP** | | | **RR** | | | **RP** | | |
| **Response** | **BL** | **T1** | **T2** | **BL** | **T1** | **T2** | **BL** | **T1** | **T2** |
| MUCH WORSE | 0 | 0 | 0 | 0 | 0 | 0 | 0 | 0 | 1 |
| SLIGHTLY WORSE | 1 | 0 | 1 | 0 | 1 | 2 | 4 | 1 | 5 |
| NO CHANGE | 28 | 13 | 14 | 27 | 9 | 6 | 30 | 15 | 13 |
| SLIGHTLY IMPROVED | 4 | 15 | 10 | 8 | 14 | 12 | 2 | 14 | 7 |
| GREATLY IMPROVED | 0 | 5 | 7 | 0 | 9 | 12 | 0 | 6 | 8 |

Supplementary Table 4: Frequency distributions of responses for ‘Quality of Life’. Frequencies provided for each group at each time point. P = placebo; R = robenacoxib; N = number of animals; Std Error = standard error; T1 = end of 3 week treatment period; T2 = end of 6 week treatment period; QoL = Quality of Life.

| **Group (treatment sequence)** | | **1 (PP)** | | | **2 (RR)** | | | **3 (RP)** | | |
| --- | --- | --- | --- | --- | --- | --- | --- | --- | --- | --- |
| **Variable** | **Time** | **n** | **Mean** | **SD** | **n** | **Mean** | **SD** | **n** | **Mean** | **SD** |
| **WBC***  **(1000/UL)** | Pre | 36 | 7.0 | 3.11 | 36 | 7.3 | 2.40 | 36 | 9.4 | 4.28 |
|  | Exit | 36 | 7.7 | 4.04 | 36 | 8.2 | 5.47 | 36 | 8.2 | 3.26 |
| **Absolute Neutrophils***  **(/ul)** | Pre | 36 | 4756.5 | 2417.37 | 36 | 4911.4 | 1976.84 | 36 | 6711.4 | 3948.28 |
|  | Exit | 36 | 5013.1 | 3351.14 | 36 | 5558.3 | 5370.46 | 36 | 5066.3 | 2749.48 |
| **Absolute Lymphocytes**  **(/ul)** | Pre | 36 | 1515.1 | 968.16 | 36 | 1724.6 | 761.21 | 36 | 1848.8 | 911.72 |
|  | Exit | 36 | 1884.0 | 1482.84 | 36 | 1904.5 | 824.96 | 36 | 2324.6 | 1295.84 |
| **%Lymphocytes***  **(%)** | Pre | 36 | 22.0 | 11.13 | 36 | 24.7 | 10.13 | 36 | 21.6 | 10.97 |
|  | Exit | 36 | 25.1 | 12.30 | 36 | 26.5 | 11.75 | 36 | 29.0 | 12.36 |
| **%Neutrophils***  **(%)** | Pre | 36 | 67.5 | 12.44 | 36 | 66.2 | 11.88 | 36 | 68.7 | 12.24 |
|  | Exit | 36 | 64.3 | 13.70 | 36 | 64.0 | 13.54 | 36 | 60.4 | 12.39 |
| **RBC**  **(MILL/CMM)** | Pre | 36 | 7.9 | 1.28 | 36 | 7.7 | 1.15 | 36 | 7.7 | 1.32 |
|  | Exit | 36 | 8.4 | 0.93 | 36 | 8.4 | 1.03 | 36 | 8.1 | 1.32 |
| **Hematocrit**  **(%)** | Pre | 36 | 38.1 | 6.76 | 36 | 37.7 | 5.10 | 36 | 38.0 | 5.85 |
|  | Exit | 36 | 40.8 | 5.28 | 36 | 40.4 | 4.93 | 36 | 39.4 | 5.81 |
| **Hemoglobin**  **(G/DL)** | Pre | 36 | 12.1 | 2.10 | 36 | 11.9 | 1.58 | 36 | 11.8 | 1.66 |
|  | Exit | 36 | 12.8 | 1.62 | 36 | 12.8 | 1.49 | 36 | 12.4 | 1.80 |
| **Platelet Count**  **(THOUS/CMM)** | Pre | 36 | 237.0 | 100.79 | 36 | 218.9 | 79.11 | 36 | 250.9 | 96.74 |
|  | Exit | 36 | 238.8 | 93.26 | 36 | 197.1 | 68.19 | 36 | 242.2 | 97.12 |

Supplementary Table 5: Select hematological values by treatment group. Pre = Screening, Day -14; Exit = Study Exit, Day 42; P = placebo; R = robenacoxib; n = number of patients; SD = Standard Deviation. *Denotes statistically significant differences (Least Squares Mean difference, p<0.05) between RP and RR groups.

| **Group (treatment sequence)** |  | **1 (PP)** | | | **2 (RR)** | | | **3 (RP)** | | |
| --- | --- | --- | --- | --- | --- | --- | --- | --- | --- | --- |
| **Variable** | **Time** | **n** | **Mean** | **SD** | **n** | **Mean** | **SD** | **n** | **Mean** | **SD** |
| **Albumin**  **(G/DL)** | Pre | 36 | 3.5 | 0.30 | 36 | 3.4 | 0.27 | 36 | 3.5 | 0.32 |
|  | Exit | 36 | 3.6 | 0.28 | 36 | 3.5 | 0.29 | 36 | 3.5 | 0.35 |
| **Globulin**  **(G/DL)** | Pre | 36 | 3.8 | 0.59 | 36 | 3.9 | 0.64 | 36 | 3.9 | 0.48 |
|  | Exit | 36 | 4.0 | 0.96 | 36 | 4.0 | 0.65 | 36 | 4.1 | 0.52 |
| **Total Protein**  **(G/DL)** | Pre | 36 | 7.3 | 0.59 | 36 | 7.3 | 0.58 | 36 | 7.5 | 0.50 |
|  | Exit | 36 | 7.6 | 0.88 | 36 | 7.5 | 0.62 | 36 | 7.6 | 0.51 |
| **Glucose**  **(MG/DL)** | Pre | 36 | 129.2 | 47.90 | 36 | 127.4 | 48.03 | 36 | 124.7 | 44.50 |
|  | Exit | 36 | 117.9 | 46.31 | 36 | 110.5 | 32.40 | 36 | 114.9 | 28.86 |
| **AST (SGOT)**  **(/UL)** | Pre | 36 | 31.6 | 14.48 | 36 | 27.5 | 7.60 | 36 | 32.7 | 10.61 |
|  | Exit | 36 | 27.1 | 8.39 | 36 | 29.2 | 9.54 | 36 | 26.8 | 7.97 |
| **ALT (SGPT)**  **(/UL)** | Pre | 36 | 56.6 | 24.42 | 36 | 53.0 | 23.40 | 36 | 61.8 | 32.87 |
|  | Exit | 36 | 54.4 | 28.33 | 36 | 52.2 | 25.71 | 36 | 50.9 | 19.77 |
| **Alkaline Phosphatase**  **(/UL)** | Pre | 36 | 23.1 | 14.16 | 36 | 24.2 | 9.22 | 36 | 24.3 | 10.19 |
|  | Exit | 36 | 25.3 | 14.81 | 36 | 26.7 | 10.77 | 36 | 25.6 | 11.66 |
| **CPK**  **(/UL)** | Pre | 36 | 377.8 | 442.72 | 36 | 347.4 | 297.14 | 36 | 419.6 | 332.64 |
|  | Exit | 36 | 195.2 | 151.45 | 36 | 335.2 | 505.92 | 36 | 235.6 | 176.58 |
| **Triglycerides**  **(MG/DL)** | Pre | 36 | 66.8 | 56.19 | 36 | 59.1 | 39.16 | 36 | 63.3 | 53.25 |
|  | Exit | 36 | 98.8 | 151.68 | 36 | 70.8 | 35.90 | 36 | 85.4 | 81.70 |
| **Lipase***  **(/UL)** | Pre | 18 | 129.6 | 52.56 | 17 | 72.2 | 40.46 | 18 | 84.8 | 80.78 |
|  | Exit | 18 | 139.6 | 57.68 | 17 | 70.8 | 37.60 | 18 | 78.3 | 74.78 |
| **Lipase (Precision PSL)**  **(/UL)** | Pre | 15 | 20.6 | 10.78 | 15 | 22.9 | 26.32 | 16 | 24.0 | 16.35 |
|  | Exit | 15 | 24.5 | 18.75 | 15 | 25.1 | 26.91 | 16 | 26.2 | 15.59 |
| **Cholesterol**  **(MG/DL)** | Pre | 36 | 186.9 | 49.23 | 36 | 173.0 | 49.92 | 36 | 161.1 | 45.66 |
|  | Exit | 36 | 192.1 | 49.75 | 36 | 171.0 | 39.04 | 36 | 172.7 | 58.48 |
| **Creatinine**  **(MG/DL)** | Pre | 36 | 1.6 | 0.38 | 36 | 1.5 | 0.30 | 36 | 1.5 | 0.37 |
|  | Exit | 36 | 1.6 | 0.46 | 36 | 1.6 | 0.29 | 36 | 1.5 | 0.43 |
| **Urea Nitrogen**  **(MG/DL)** | Pre | 36 | 27.8 | 7.28 | 36 | 27.2 | 6.21 | 36 | 28.3 | 7.94 |
|  | Exit | 36 | 28.8 | 7.83 | 36 | 28.8 | 7.41 | 36 | 28.6 | 7.24 |
| **Magnesium**  **(MEQ/L)** | Pre | 36 | 1.9 | 0.23 | 36 | 1.9 | 0.22 | 36 | 1.9 | 0.23 |
|  | Exit | 36 | 2.0 | 0.18 | 36 | 2.0 | 0.21 | 36 | 2.0 | 0.17 |
| **Phosphorus**  **(MEQ/L)** | Pre | 36 | 4.2 | 0.78 | 36 | 4.1 | 0.63 | 36 | 4.4 | 0.69 |
|  | Exit | 36 | 4.3 | 0.71 | 36 | 4.4 | 0.78 | 36 | 4.5 | 0.64 |
| **Potassium**  **(MEQ/L)** | Pre | 36 | 4.3 | 0.46 | 36 | 4.2 | 0.48 | 36 | 4.3 | 0.41 |
|  | Exit | 36 | 4.4 | 0.43 | 36 | 4.4 | 0.46 | 36 | 4.4 | 0.40 |
| **Sodium**  **(MEQ/L)** | Pre | 36 | 151.8 | 1.92 | 36 | 152.2 | 1.95 | 36 | 152.1 | 2.23 |
|  | Exit | 36 | 153.1 | 3.22 | 36 | 153.4 | 3.57 | 36 | 153.0 | 2.26 |
| **Chloride**  **(MEQ/L)** | Pre | 36 | 118.8 | 2.49 | 36 | 119.0 | 2.54 | 36 | 117.9 | 3.25 |
|  | Exit | 36 | 118.4 | 3.65 | 36 | 118.9 | 2.93 | 36 | 117.4 | 2.80 |

Supplementary Table 6: Select chemistry values by treatment group. Pre = Screening, Day -14; Exit = Study Exit, Day 42; P = placebo; R = robenacoxib; n = number of patients; SD = Standard Deviation. * Denotes statistically significant differences (Least Squares Mean difference, *P* < 0.05) between PP and RP, and between PP and RR groups.

| **Group (treatment sequence)** |  | **1 (PP)** | | | **2 (RR)** | | | **3 (RP)** | | |
| --- | --- | --- | --- | --- | --- | --- | --- | --- | --- | --- |
| **Variable** | **Time** | **N** | **Mean** | **SD** | **N** | **Mean** | **SD** | **N** | **Mean** | **SD** |
| **Specific Gravity** | Pre | 35 | 1.0 | 0.02 | 36 | 1.0 | 0.01 | 36 | 1.0 | 0.02 |
|  | Exit | 35 | 1.0 | 0.02 | 36 | 1.0 | 0.02 | 36 | 1.0 | 0.02 |
| **pH** | Pre | 35 | 6.5 | 0.46 | 36 | 6.5 | 0.40 | 36 | 6.6 | 0.56 |
|  | Exit | 35 | 6.8 | 0.80 | 36 | 6.7 | 0.58 | 36 | 6.7 | 0.81 |

Supplemental Table 7: Select urinalysis results by treatment group. Pre = Screening, Day -14; Exit = Study Exit, Day 42; P = placebo; R = robenacoxib; n = number of patients; SD = Standard Deviation.

**Quality of Life**

**Compared to before your cat received this most recent treatment,**

do you think your cat’s **overall quality of life** is (check one):

| Much worse |  |
| --- | --- |
| Slightly worse |  |
| No change |  |
| Slightly improved |  |
| Greatly improved |  |

**Temperament**

**Compared to before your cat received this most recent treatment do you think your cat’s temperament** (mood, demeanor) **is**:

| Much worse |  |
| --- | --- |
| Slightly worse |  |
| No change |  |
| Slightly improved |  |
| Greatly improved |  |

**Happiness**

**Compared to your last visit, would you say your cat is:**

| Much more unhappy |  |
| --- | --- |
| Slightly more unhappy |  |
| No change in happiness |  |
| Slightly more happy |  |
| Much more happy |  |

Supplemental Figure 1: Patient Quality of Life (QoL), temperament, and happiness owner assessment metrology instruments.
